# Role of Glucose-6-Phosphate in Metabolic Adaptation of Staphylococcus aureus in Diabetes

**DOI:** 10.1128/Spectrum.00857-21

**Published:** 2021-09-22

**Authors:** Keun Seok Seo, Nogi Park, Jaime K. Rutter, Youngkyung Park, Carol L. Baker, Justin A. Thornton, Joo Youn Park

**Affiliations:** a Department of Comparative Biomedical Sciences, College of Veterinary Medicine, Mississippi State University, Mississippi State, Mississippi, USA; b Department of Biological Sciences, College of Arts and Sciences, Mississippi State University, Mississippi State, Mississippi, USA; Emory University School of Medicine

**Keywords:** *Staphylococcus aureus*, diabetes, glucose-6-phosphate, hexose phosphate transport system, bicomponent leukocidins

## Abstract

Diabetic foot ulcer (DFU) is the most common and costly sequela of diabetes mellitus, often leading to lower-extremity amputation with poor 5-year survival rates. Staphylococcus aureus is the most prevalent pathogen isolated from DFU, suggesting adaptation of S. aureus to the unique metabolic conditions of diabetes. Diabetes is a complex metabolic disorder with increases not only in serum glucose levels but also in levels of other sugars, including fructose, mannose, and glucose-6-phosphate (G6P). However, the effect of metabolism of these sugars on the pathogenesis of S. aureus is not fully understood. In this study, we demonstrated that metabolism of G6P, fructose, and mannose induced greater expression of staphylococcal virulence factors than did glucose metabolism, but only G6P effects were independent of glucose-mediated carbon catabolite repression, suggesting a physiologically relevant role in diabetes. Our *in vivo* studies further demonstrated that G6P was highly present in skin adipose tissues of diabetic TALLYHO/JngJ mice, and subcutaneous infection with S. aureus caused significantly greater tissue necrosis and bacterial burden, compared to nondiabetic SWR/J mice. Finally, enhanced pathogenesis of S. aureus in diabetic TALLYHO/JngJ mice was significantly attenuated by deletion of the hexose phosphate transport (HPT) system. These results suggest that G6P is an important metabolic signal for S. aureus, enhancing the virulence in diabetes. A better understanding of how G6P metabolism is linked to the virulence of S. aureus will lead to the development of novel alternative therapeutics.

**IMPORTANCE** Sugars are essential nutrients for S. aureus to survive and proliferate within the host. Because elevated serum glucose levels are a hallmark of diabetes, most studies have focused on the effect of glucose metabolism, and very little is known regarding the effects of metabolism of other sugars on the pathogenesis of S. aureus in diabetes. In this study, we demonstrated that G6P, which is highly present in diabetes, can induce expression of staphylococcal virulence factors that cause severe tissue necrosis and bacterial burden in skin infections. Our results highlight the importance of nutritional control of blood sugar levels, not only glucose but also other highly metabolizable sugars such as G6P. A better understanding of how activation of the HPT system is linked to the virulence of S. aureus will guide development of novel alternative therapeutics.

## INTRODUCTION

Diabetes mellitus is a chronic metabolic disorder characterized by abnormally high blood sugar levels resulting from impairment of insulin production. This is driven by either defective beta cells of the pancreas (type 1 diabetes) or insulin resistance caused by multiple factors, including obesity, genetics, and lifestyle (type 2 diabetes [T2D]). T2D accounts for 90 to 95% of all diagnosed cases of diabetes. Nearly 350 million people were diagnosed with T2D worldwide in 2013, and the number is anticipated to be more than 500 million by 2035 (1). Among several complications associated with diabetes are vascular disease, renal failure, blindness, heart failure, and diabetic foot ulcer (DFU), with DFU being the most prevalent and significant sequela of T2D. The total economic loss caused by diagnosed diabetes in the United States in 2017 was estimated to be $327 billion, including $237 billion in direct medical costs and $90 billion in reduced productivity (2). It is estimated that 19 to 34% of patients with T2D develop DFU in their lifetimes, and DFU is the most common cause of hospitalization and medical costs associated with diabetes (3). Despite high health care costs, outcomes for patients presenting with DFU infections are poor; such infections often result in lower-limb amputation, with very poor 1-, 2-, and 5-year survival rates of 80.80%, 69.01%, and 28.64%, respectively (4). DFU is caused by a combination of peripheral sensorimotor and autonomic neuropathy, peripheral vascular disease, and minor trauma, frequently complicated by subsequent infections (5). Several metagenomic studies demonstrated that Staphylococcus aureus is the most common pathogen isolated from DFU infections (6–9). Furthermore, microbiome studies demonstrated that patients with T2D showed skin microbiota more frequently colonized with S. aureus and more susceptible to S. aureus infections (6–9). These findings suggest that S. aureus successfully adapts to the diabetic condition; however, the underlying mechanism has not been fully elucidated.

S. aureus produces a battery of virulence factors to colonize, invade, and destroy tissues and components of the host immune system (9–11). These virulence factors include microbial surface components recognizing adhesive matrix molecules promoting colonization, staphylococcal cytolytic proteins directly killing host immune cells, and staphylococcal superantigens aberrantly activating immune cells to cause a cytokine storm (12, 13). Expression of these virulence factors is mainly regulated by regulatory two-component systems (TCSs), such as the S. aureus exoprotein expression (Sae) TCS and the accessory gene regulator (Agr) TCS, in response to a quorum-sensing signal that is dependent on the population density, as well as external signals such as oxygen, pH, changes in osmolality, and host-driven antimicrobial peptides (14, 15). In a DFU, S. aureus is exposed to unique metabolic conditions in diabetes. Among them, glucose is the most significant metabolic cue, to which S. aureus must swiftly respond in order to produce energy and cellular components related to virulence. Previous studies demonstrated that metabolism of glucose contributes to the pathogenesis of S. aureus by promoting biofilm formation, resistance to NO^−^, and replication within tissues (16–18). In contrast, other studies demonstrated that metabolism of glucose suppressed the pathogenesis of S. aureus by repressing expression of Agr-dependent virulence factors, including α-hemolysin, β-hemolysin, protein A, and staphylococcal enterotoxin B (19–22). These toxins are key virulence factors of S. aureus that disarm the host immune system. However, it is not clearly understood how S. aureus achieves successful pathogenesis in the presence of elevated glucose levels, which suppress expression of staphylococcal virulence factors.

Recent progress in metabolomic studies revealed that diabetes results not only in elevated serum glucose levels but also in many other metabolites, including several sugars such as fructose, mannose, and glucose-6-phosphate (G6P) (23–26). Recently, we demonstrated that metabolism of G6P significantly increased survival/multiplication of S. aureus within host innate immune cells and resistance to antibiotics (27). These results suggest that metabolism of other sugars may affect the pathogenesis of S. aureus, which has not been fully elucidated. In this study, we investigated how metabolism of sugars elevated during diabetes impacts the pathogenesis of S. aureus. We demonstrate that G6P is an important metabolic signal in diabetes that promotes the pathogenesis of S. aureus by inducing expression of staphylococcal cytotoxins, leading to severe tissue necrosis and bacterial burdens in diabetic TALLYHO/JngJ mice.

## RESULTS

### Metabolism of sugars highly present in diabetes induces expression of staphylococcal virulence factors.

**FIG 1:**
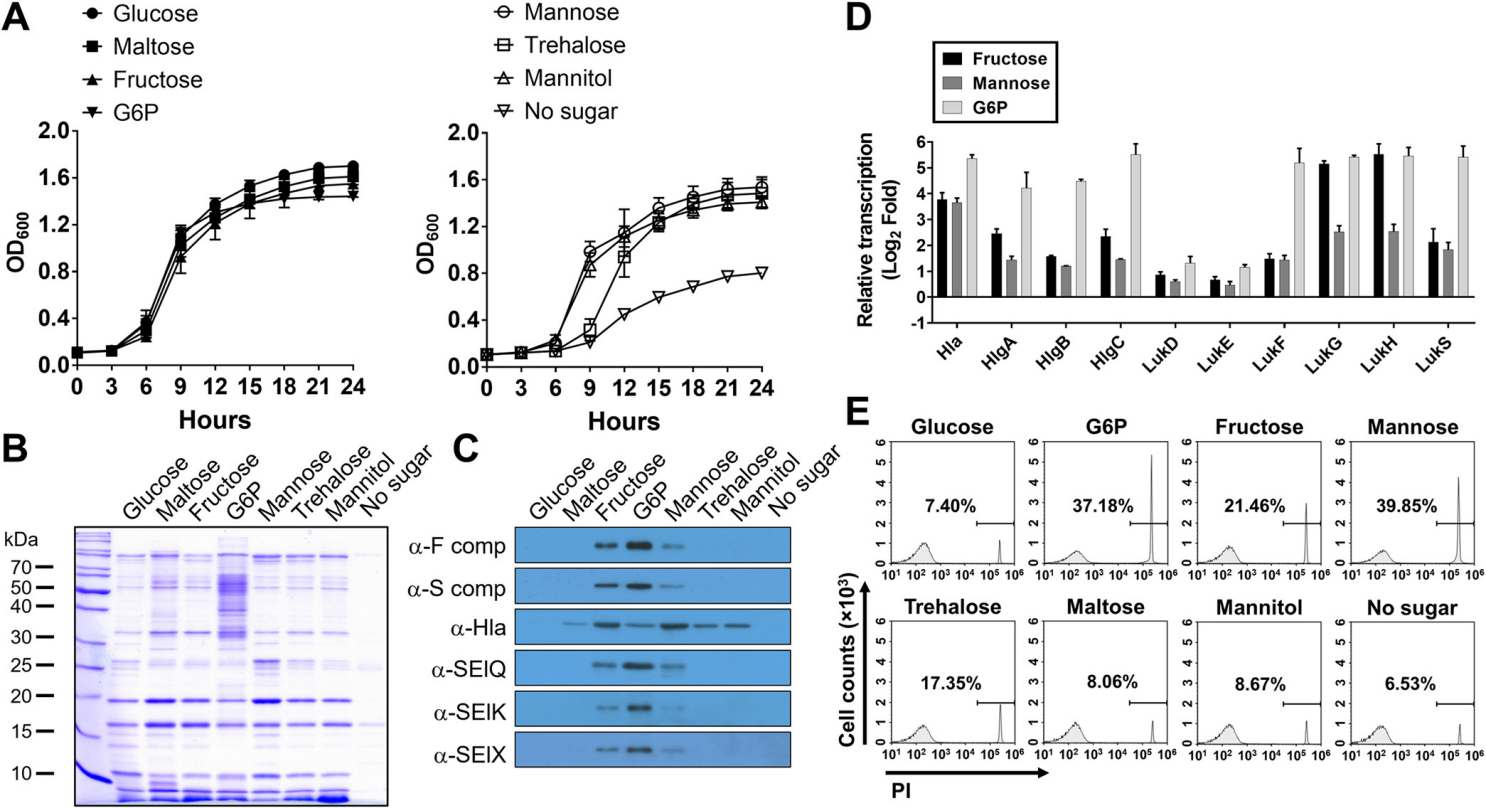
Effect of sugar metabolism on expression of staphylococcal virulence factors. The S. aureus LAC strain was cultured in CDM supplemented with a single sugar (glucose, maltose, fructose, G6P, mannose, trehalose, or mannitol). (A) Growth at 37°C for 24 h was analyzed by measuring the optical density at 600 nm. (B) Exoprotein profiles of culture supernatants prepared by TCA precipitation were analyzed by SDS-PAGE. (C) Expression of staphylococcal virulence factors was analyzed by immunoblotting using antibodies specific to staphylococcal cytotoxins and enterotoxins. (D) Transcription of staphylococcal virulence factors was analyzed by qRT-PCR. The fold change was calculated by comparing the *C_T_* normalized to the internal control (*gyrA*), relative to the results with glucose. (E) Cytotoxicity of culture supernatants was measured by incubation with human whole leukocytes (2 × 10^5^ cells) for 1 h. Cytotoxicity, indicated by incorporation of PI into cellular DNA, was measured using a NovoCyte flow cytometer. Representative results are shown. All experiments were repeated three times. Error bars indicate the standard deviations.

To determine the effect of sugar metabolism on the expression of staphylococcal virulence factors, S. aureus strain LAC, a common genotype of S. aureus isolated from skin and soft tissue infections in the United States, was cultured in chemically defined medium (CDM) supplemented with a single sugar, i.e., glucose, fructose, mannitol, trehalose, mannose, maltose, or G6P, each highly present in the serum and tissues of T2D (23–26). S. aureus LAC grew at similar rates in CDM supplemented with all tested sugars except trehalose, with which S. aureus entered the early exponential phase of growth approximately 3 h later than with the other sugars but reached the stationary phase at 24 h of culture, similar to findings for the other sugars (Fig. 1A). In contrast, exoprotein expression profiles were highly altered by metabolism of each specific sugar (Fig. 1B). Immunoblot analysis showed that expression of bicomponent leukocidins (F and S components) and staphylococcal enterotoxins (SElQ, SElK, and SElX) was highly induced in the cultures in CDM supplemented with G6P, followed by fructose and mannose (Fig. 1C). Expression of α-hemolysin was broadly induced in CDM supplemented with all tested sugars except glucose. In contrast, staphylococcal virulence factors were least expressed in CDM supplemented with glucose. Consistent with immunoblot results, quantitative real-time PCR (qRT-PCR) analysis showed that transcription of staphylococcal cytotoxins was highly induced in cultures in CDM supplemented with G6P, fructose, or mannose (2- to 34-fold higher than with glucose), compared to CDM supplemented with glucose (Fig. 1D). To confirm the expression of staphylococcal cytotoxins, cytotoxicity toward human whole leukocytes caused by culture supernatants was measured. Strong cytotoxicity was induced in the culture supernatants from CDM supplemented with G6P, followed by fructose and mannose. In contrast, no cytotoxicity was observed in culture supernatants from cells cultured with glucose (Fig. 1E).

### G6P induces expression of staphylococcal cytotoxins by activation of Agr TCS and Sae TCS independent of glucose-mediated carbon catabolite repression.

**FIG 2:**
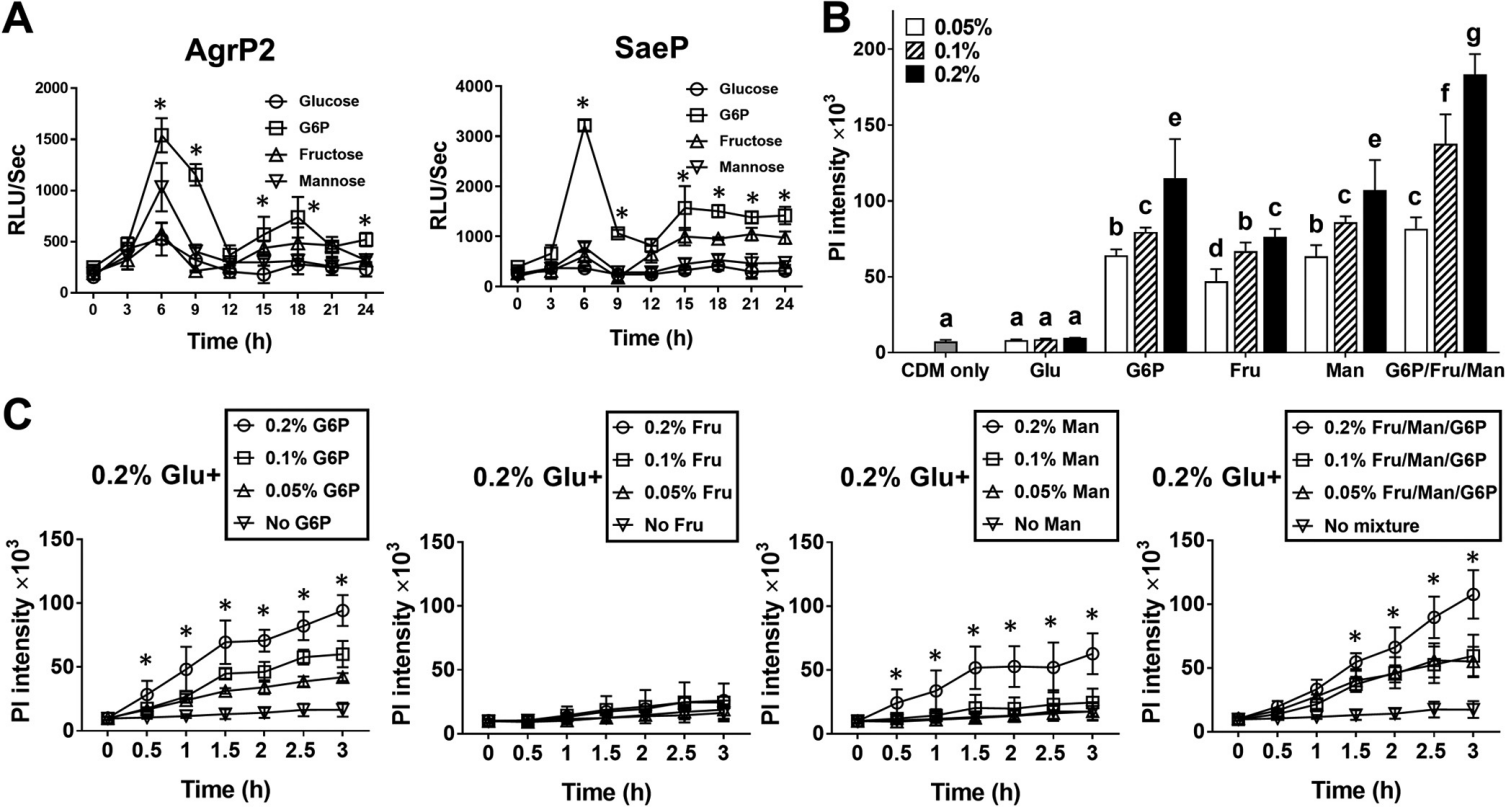
Activation of Agr and Sae regulatory TCSs by metabolism of G6P, independent of CCR by glucose. (A) S. aureus LAC luciferase reporter strains, whose expression is controlled by the promoter activity of *agr* and *sae* genes, were cultured in CDM supplemented with a single sugar (glucose, fructose, G6P, or mannose), and luciferase activity was monitored with a Cytation 5 reader. (B and C) S. aureus LAC strain was cultured for 24 h in CDM supplemented with increasing concentrations (0, 0.05, 0.1, and 0.2%) of G6P, fructose, mannose, or a mixture of all three sugars (B) or 0.2% glucose and increasing concentrations (0, 0.05, 0.1, and 0.2%) of G6P, fructose, mannose, or a mixture of all three sugars (C). Culture supernatants were incubated with human whole leukocytes (2 × 10^5^ cells/well) for 3 h. The cytotoxicity caused by culture supernatants was assessed by measuring the fluorescence signal at 590 nm caused by incorporation of PI into cellular DNA using a Cytation 5 reader. Results shown are the mean ± standard deviation from three independent experiments. Letters indicate statistical significance analyzed by ANOVA and Tukey’s multiple-comparison test (*P* < 0.05). *, Statistical significance, compared to the results from CDM supplemented with glucose, by Student's *t* test (*P* < 0.05).

To determine the extent to which the expression of staphylococcal virulence factors induced by metabolism of fructose, mannose, or G6P resulted from transcriptional activation of Agr and Sae, we generated S. aureus LAC strain harboring a luciferase reporter plasmid (28) whose expression is controlled by the promoter activity of Agr and Sae. Significantly greater transcriptional activity of both Agr and Sae was observed from CDM supplemented with G6P, compared to results from CDM supplemented with glucose, which did not induce transcriptional activation of Agr or Sae (Fig. 2A). These results suggest that metabolism of G6P induced transcriptional activation of Agr and Sae, which led to expression of staphylococcal virulence factors.

Carbon catabolite repression (CCR) is a mechanism by which the presence of a preferred carbon source inhibits the expression of genes and functions related to the use of secondary carbon sources. This mechanism allows bacteria to selectively use the preferred carbon source from a mixture of different carbon sources (29). Since glucose is generally the most preferable sugar for many bacteria (30) and glucose concentrations are highly elevated in diabetes, it is possible that induction of staphylococcal virulence factors by G6P might be abrogated due to CCR in the presence of glucose, thereby not being physiologically relevant to diabetes. To test this possibility, S. aureus LAC strain was cultured in CDM alone or supplemented with increasing concentrations of G6P, fructose, or mannose in the absence or presence of 0.2% (wt/vol) glucose, a glucose level similar to that seen in diabetes. Supplementation with G6P, fructose, mannose, or a mixture of these sugars significantly increased cytotoxicity, in a dose-dependent manner (Fig. 2B). Addition of glucose to the medium significantly decreased cytotoxicity caused by growth on fructose and mannose (with an exception at 0.2% mannose), while cytotoxicity caused by growth on G6P was not inhibited (Fig. 2C). These results indicated that the expression of staphylococcal virulence factors induced by G6P is independent of CCR caused by glucose, suggesting that G6P might have a physiologically relevant role in diabetes. Based on these findings, we focused on the role of G6P in the pathogenesis of S. aureus in diabetes.

### Host intracellular G6P induces expression of staphylococcal cytotoxins that is dependent on the HPT system.

**FIG 3:**
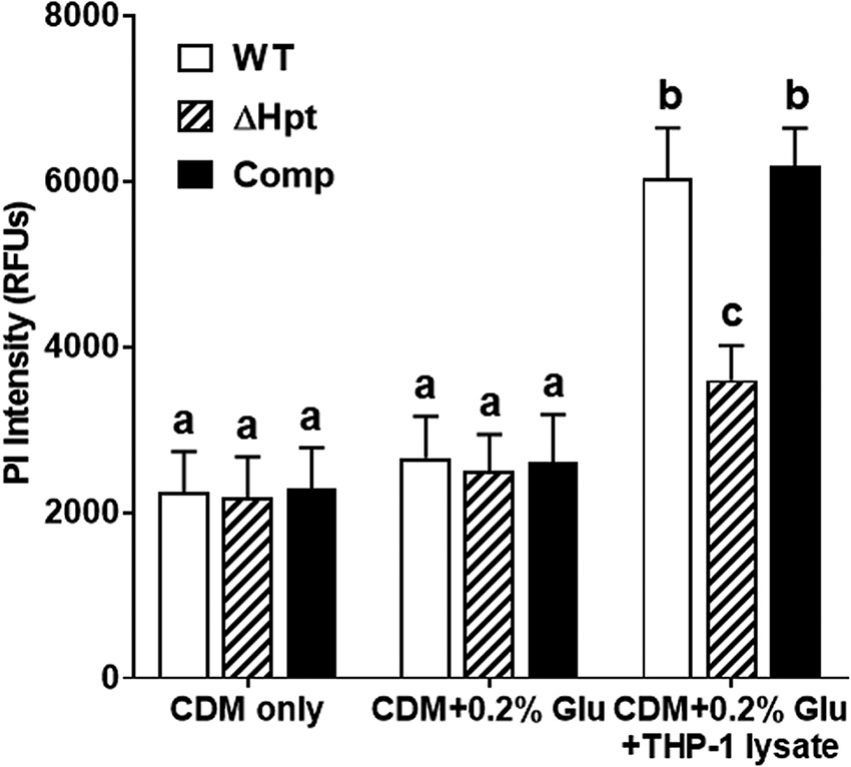
Host intracellular G6P induces expression of staphylococcal cytotoxins that is dependent on the HPT system. S. aureus LAC wild-type (WT), ΔHpt, or complemented strains were cultured in CDM alone, CDM supplemented with 0.2% glucose, or CDM supplemented with 0.2% glucose and THP-1 cell lysate. Culture supernatants were incubated with human whole leukocytes (2 × 10^5^ cells/well) for 1 h. The cytotoxicity caused by culture supernatants was measured using a Cytation 5 reader. Lower case letters indicate statistical significance analyzed using ANOVA and Tukey’s multiple-comparison test (*P* < 0.05).

The majority of G6P within the host is intracellular, because glucose is transported into cells through a glucose transporter (GLUT) and converted to G6P by hexokinase to prevent diffusion out of cells. To test whether G6P present in the host is sufficient to induce expression of staphylococcal cytotoxins, we first measured the intracellular concentration of G6P in THP-1 monocytes, representing the primary target of staphylococcal cytotoxins. We found approximately 17.2 ± 0.84 μM G6P (0.442% [wt/vol]) in THP-1 cell lysates prepared from 1 × 10^7^ cells. Supplementation of THP-1 lysates into CDM containing 0.2% glucose induced significantly greater cytotoxicity of the S. aureus LAC strain than without supplementation (Fig. 3). Since THP-1 lysates contained not only G6P but also many other cellular components, cytotoxicity might be induced by other cellular components. To determine whether G6P in the THP-1 cell lysates was responsible for the induction of cytotoxicity, we generated a S. aureus LAC strain lacking the hexose phosphate transport (HPT) system (*hptARS* and *uhpT* genes) (ΔHpt), which is unable to respond to extracellular G6P. Disruption of the HPT system significantly reduced cytotoxicity induced by supplementation with THP-1 lysates, which was restored by complementation of the HPT system (Fig. 3). Combined, these results indicated that intracellular G6P is responsible for inducing the expression of staphylococcal virulence factors.

### S. aureus causes severe pathology in diabetic TALLYHO/JngJ mice, which is dependent on the HPT system.

**FIG 4:**
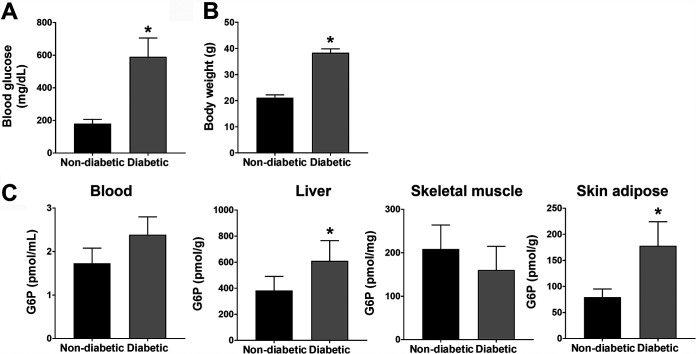
Quantification of G6P in tissue samples from diabetic TALLYHO/JngJ mice and nondiabetic SWR/J mice. (A and B) Male TALLYHO/JngJ mice spontaneously developed hyperglycemia (A) and mild obesity (B) at the age of 12 weeks. (C) Diabetic TALLYHO/JngJ mice showed significantly higher concentrations of G6P in liver and skin adipose tissue, compared to nondiabetic SWR/J mice. Results shown are the mean ± standard deviation from three independent experiments. *, Statistical significance using Student's *t* test (*P* < 0.05).

To investigate the role of G6P in the pathogenesis of S. aureus *in vivo*, we used male TALLYHO/JngJ mice, which display the polygenic etiology of diabetes, spontaneously developing hyperglycemia, hyperlipidemia, hyperinsulinemia, and moderate obesity at the age of 12 weeks; this mimics many characteristics of T2D in humans (13, 14). We first investigated whether TALLYHO/JngJ diabetic mice have elevated G6P levels in tissues, as observed in humans. After spontaneously developing hyperglycemia and moderate obesity at the age of 12 weeks (Fig. 4A and B), TALLYHO/JngJ diabetic mice showed significantly higher concentrations of G6P in skin adipose tissues and liver, compared with nondiabetic SWR/J control mice (Fig. 4C).

**FIG 5:**
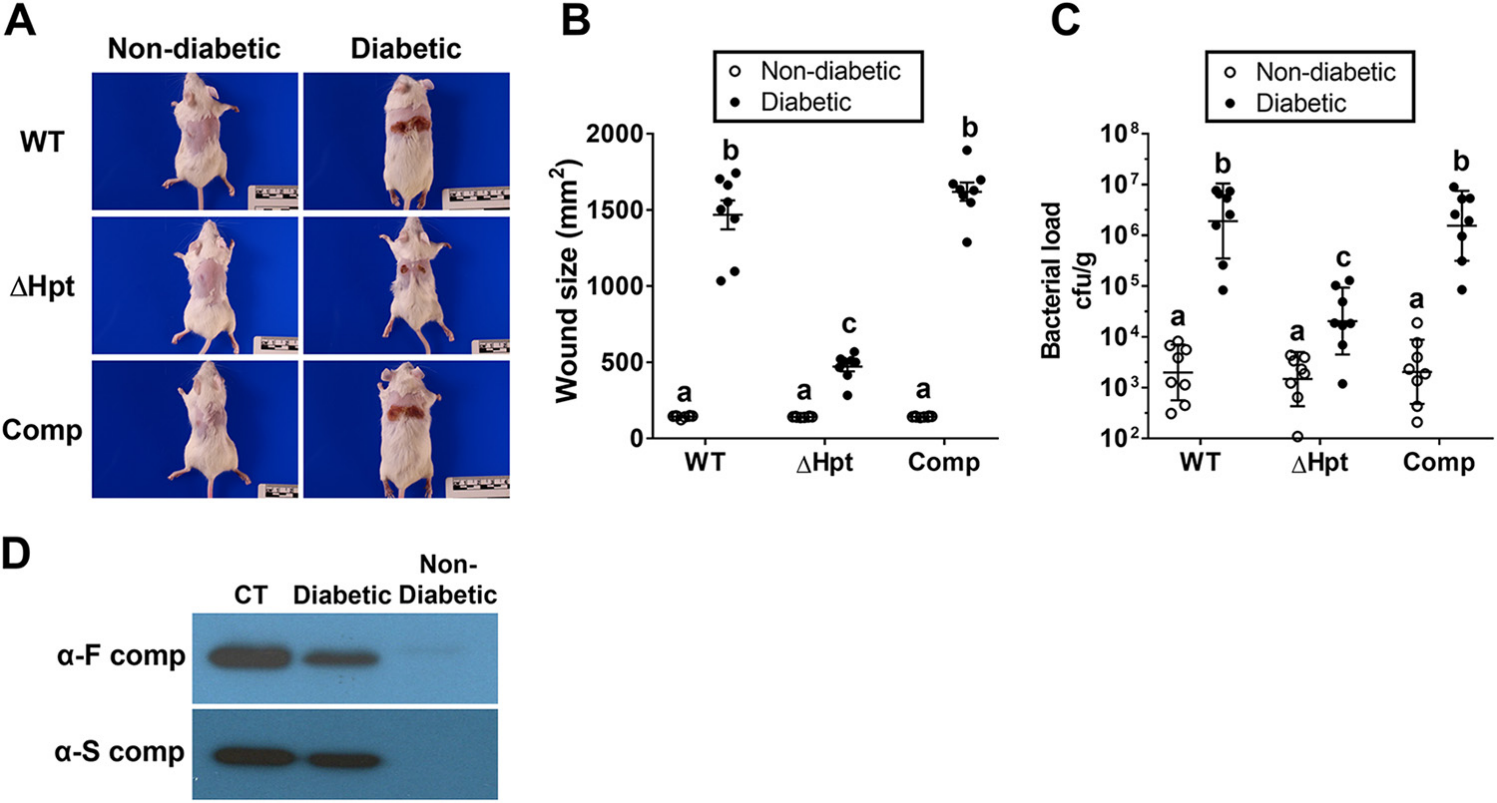
S. aureus causes severe pathology in diabetic TALLYHO/JngJ mice, which is dependent on the HPT system. Diabetic TALLYHO/JngJ and nondiabetic SWR/J mice (*n* = 4/strain in two independent experiments) were subcutaneously infected with S. aureus LAC wild-type (WT), ΔHpt, or complemented strains (1 × 10^6^ CFU). (A and B) After 10 days, animals were humanely euthanized (A), and the areas of skin lesions were measured using an ARANZ SilhouetteStar camera (B). (C) The number of bacteria existing in the skin lesions was analyzed. Results shown are the mean ± standard deviation. Statistical significance was analyzed using ANOVA and Tukey’s multiple-comparison test (*P* < 0.05). (D) Expression of staphylococcal cytotoxins in the skin lesions was analyzed using immunoblotting. Purified LukF and LukS were used as F-component and S-component controls, respectively. CT, control.

The higher G6P levels in skin adipose tissues of TALLYHO/JngJ diabetic mice was intriguing since S. aureus frequently colonizes skin and causes skin infections in diabetes, often progressing to severe DFU. To determine the role of elevated G6P levels in diabetic skin adipose tissues in the virulence of S. aureus, age-matched diabetic TALLYHO/JngJ mice and nondiabetic SWR/J control mice were subcutaneously infected with S. aureus LAC wild-type, ΔHpt, or complemented strains. Infection with the S. aureus LAC wild-type strain caused significant, severe tissue necrosis and bacterial burden in diabetic TALLYHO/JngJ mice, compared to nondiabetic SWR/J control mice (Fig. 5A to C). Disruption of the HPT system (ΔHpt strain) significantly attenuated the virulence of S. aureus in diabetic TALLYHO/JngJ mice, which was restored by complementation of the HPT system. In contrast, deletion of the HPT system did not affect the virulence of S. aureus in nondiabetic SWR/J control mice. Expression of staphylococcal cytotoxins in the lesions was analyzed using Western blotting. There was strong expression of the bicomponent leukocidins in the lesions from the diabetic TALLYHO/JngJ mice but not in those from nondiabetic SWR/J control mice (Fig. 5D), while expression of α-hemolysin was not detected in either group (data not shown). These results indicated that S. aureus causes severe pathology in diabetic TALLYHO/JngJ mice that is dependent on the HPT system responding to G6P, thus inducing expression of the bicomponent leukocidins.

### Staphylococcal cytotoxins cause epithelial cell death indirectly by lysing host neutrophils.

**FIG 6:**
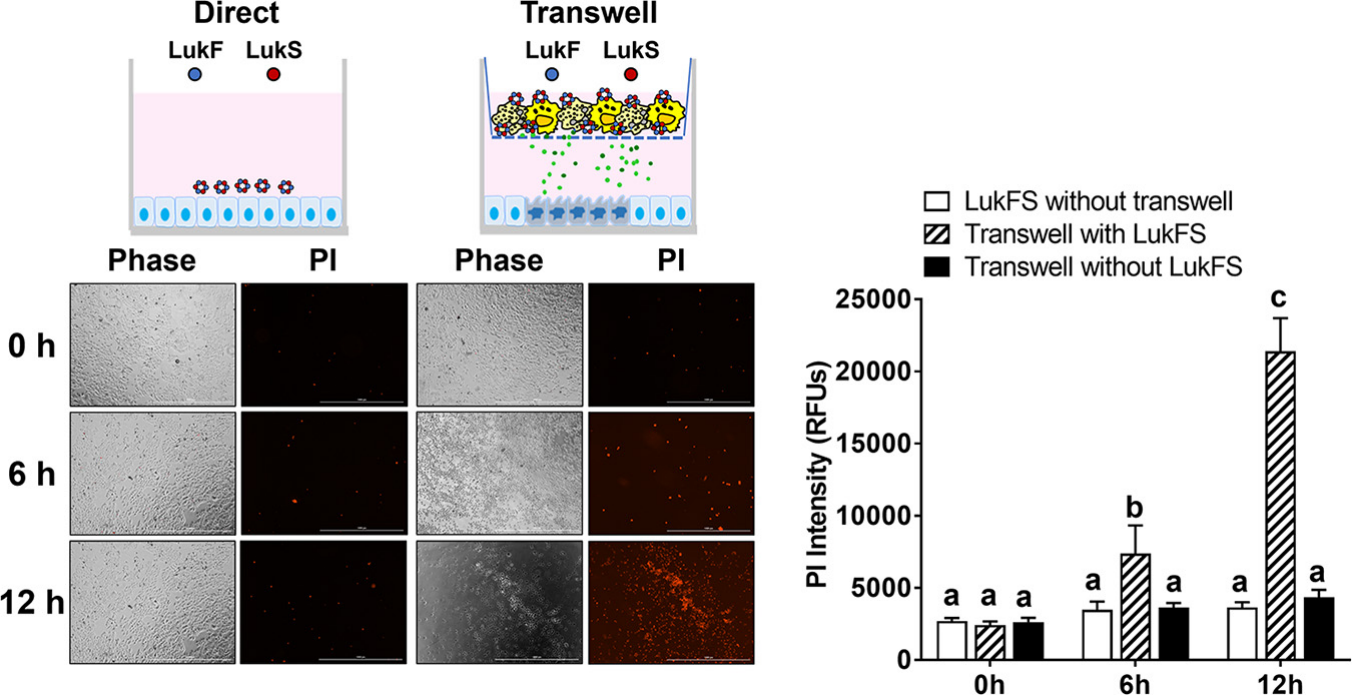
Lysis of host neutrophils by staphylococcal cytotoxins causes epithelial cell death. Human HEp2 cells were cultured in a 24-well plate to 80% confluence. A 0.45-μm Transwell insert containing human neutrophils at 1 × 10^5^ cells/well was placed in the 24-well plate. LukF and LukS (1 μg each) were added to the Transwells, and cells were incubated for up to 12 h. As controls, HEp2 cells were incubated with Transwells only (without LukF and LukS) or incubated with LukF and LukS without Transwells. Cytotoxicity was assessed by taking images or measuring fluorescence signals at 590 nm using a Cytation 5 reader (BioTek). Representative images are shown. Lower case letters indicate statistical significance analyzed using ANOVA and Tukey’s multiple-comparison test (*P* < 0.05).

Although the bicomponent leukocidins were highly expressed in lesions of diabetic TALLYHO/JngJ mice, their contribution to the development of tissue necrosis is not clear because bicomponent leukocidins are not toxic to epithelial cells, due to the lack of cytotoxin receptors on epithelial cells. Since bicomponent leukocidins are highly toxic to neutrophils and neutrophils can cause tissue damage by releasing proinflammatory cytokines, reactive oxygen species, neutrophil extracellular traps, and metalloproteases, we questioned whether severe tissue necrosis caused by S. aureus infection resulted from the lysis of neutrophils by staphylococcal cytotoxins. To test this possibility, human neutrophils were placed in Transwell inserts with 0.45-μm porous membranes, which allow diffusion of small molecules released from lysis of neutrophils by staphylococcal cytotoxins to HEp2 epithelial cells grown in the bottom of the wells. After 6 h of LukF/S treatment to the Transwells, HEp2 epithelial cells were detached and rounded but were negative for propidium iodide (PI) staining (Fig. 6). After 12 h, HEp2 epithelial cells became PI positive, indicating membrane damage and cell death. In contrast, the addition of LukF/S directly to the HEp2 epithelial cells or incubation with neutrophils in Transwells without LukF/S treatment did not affect the viability of HEp2 epithelial cells. These results suggest that tissue necrosis during S. aureus infection likely results from bicomponent leucocidin-dependent lysis of host neutrophils, which releases toxic effector molecules to cause epithelial cell death.

## DISCUSSION

Diabetes is a complex metabolic disorder that alters not only glucose levels but also the levels of many other metabolites (23–26, 31). G6P is a highly metabolizable sugar that impacts both host and pathogen. In hosts, glucose is transported into the cell and subsequently phosphorylated to G6P by glucokinase. Conversion of G6P to 6-phosphogluconate by G6P dehydrogenase (G6PD) provides NADPH, the principal source of intracellular reductant to prevent oxidative stress during glucose metabolism. Genetic defects in G6PD activity or excessive influx of glucose decreases intracellular NADPH levels, which in turn increases oxidative stress to cells and tissues and results in the onset of T2D and higher intracellular G6P levels (24–26). In bacteria, intracellular G6P is converted into fructose-6-phosphate, which either enters the glycolysis pathway for energy synthesis or is further converted into glucosamine-6-phosphate by the glutamine-fructose-6-phosphate amidotransferase GlmS for cell wall synthesis. G6P also can activate global gene regulatory systems in *Listeria*, Escherichia coli, Salmonella, and S. aureus that enhance intracellular survival and resistance to innate immune cells and antibiotics (32, 33). In this study, we demonstrated for the first time that G6P, which is significantly elevated in diabetes, induced expression of staphylococcal cytotoxins, causing lysis of host neutrophils, which in turn resulted in severe tissue necrosis in the diabetic host.

S. aureus encounters G6P from two different sources. One source is the conversion of glucose to G6P during the importing of extracellular glucose through the phosphotransferase system. The other source is direct uptake of extracellular G6P through the hexose phosphate transporter (UhpT) by activation of the HPT system. Previously, we characterized a three-component regulatory system, HptARS, in S. aureus (27) in which extracellular G6P is recognized by a sensor protein (HptA) that induces phosphorylation of a sensor histidine kinase (HptS), followed by phosphorylation of a response regulator (HptR). Phosphorylated HptR binds to the promoter of the *uhpT* gene and induces expression of UhpT, facilitating utilization of extracellular G6P. Our results demonstrated that staphylococcal virulence factors were induced not by glucose but rather by G6P, which was abrogated by deletion of the HPT system. These results suggest that not only the metabolism of G6P but also the activation of HptARS by extracellular G6P may impact activation of the Agr TCS and Sae TCS for global regulation of S. aureus virulence factors. We are currently investigating this possibility by using HptARS, UhpT, Agr, and Sae mutant strains and RNA sequencing analysis.

There are many validated animal models of diabetes in various species, including nonhuman primates, rabbits, rats, and mice (34). By far the most common animal models of diabetes are monogenic and polygenic murine models. The monogenic murine models such as Lep^ob/ob^ and Lepr^db/db^ mice result from a mutation in the leptin receptor gene, leading to hyperphagia, hyperglycemia, and extreme obesity, which more closely resembles the phenotype of type 1 diabetes (35). The polygenic murine models, such as male TALLYHO/JngJ mice, more closely recapitulate the phenotypes of T2D in humans, including hyperglycemia, hyperlipidemia, hyperinsulinemia, and moderate obesity (36). Since 90 to 95% of people with diabetes have T2D, we investigated the role of G6P in the pathogenesis of S. aureus in T2D *in vivo* using diabetic TALLYHO/JngJ mice. Our results demonstrated that diabetic TALLYHO/JngJ mice showed significantly higher concentrations of G6P in the skin adipose tissue and developed more severe subcutaneous infections with S. aureus, compared to nondiabetic SWR/J mice. This increase in G6P levels was significantly attenuated by disruption of the HPT system. These results demonstrate that TALLYHO/JngJ diabetic mice have similar metabolic conditions as human T2D, which makes them a useful animal model for investigating the pathogenesis of S. aureus in diabetes.

A previous study demonstrated that infection with S. aureus caused dermonecrosis, which was largely dependent on expression of α-hemolysin (37). However, S. aureus infection in diabetic TALLYHO/JngJ mice caused severe tissue necrosis without expression of detectable levels of α-hemolysin. Instead, there was strong expression of the bicomponent leukocidins. Our Transwell experiments demonstrated that lysis of neutrophils by bicomponent leukocidins caused sequential morphological changes in epithelial cells, including cell shrinkage, dissociation, detachment, and membrane damage, which could result from protease activity. A lytic form of neutrophil death induces uncontrolled release of granular proteins, oxidants, neutrophil serine proteases, and matrix metalloproteases (MMPs), which degrade extracellular matrix proteins and basement proteins to exacerbate inflammation and delay wound healing. In addition, a previous study also showed higher concentrations of MMPs (MMP-2, 9, and 8) and reduced concentrations of tissue MMP inhibitors in diabetic wounds, compared with traumatic wounds (38, 39). These findings suggest that excessive protease activity derived from lysis of neutrophils by bicomponent leukocidins may severely exacerbate inflammation and tissue injury in DFU.

In summary, we demonstrated that G6P is an important metabolic signal in diabetes that promotes the virulence of S. aureus by inducing the expression of staphylococcal virulence factors through activation of the HPT, Agr, and Sae systems. A better understanding of the mechanism by which G6P activates HptARS, Agr, and Sae systems and the key effector protease molecules released from lysis of host neutrophils by bicomponent leukocidins causing tissue necrosis will aid in the development of new therapeutics that can be tested *in vivo* with the TALLYHO/JngJ diabetic mice model established in this study.

## MATERIALS AND METHODS

### Ethics statement.

Human blood was obtained from healthy volunteers, with informed written consent. All methods used in this study were carried out in accordance with approved guidelines, and all experimental protocols were approved by the Institutional Review Board for Human Subjects at Mississippi State University (protocol 18-283). All animal experiments were reviewed and approved by the Institutional Animal Care and Use Committee at Mississippi State University (protocol 17–217). All experiments were carried out in accordance with NIH guidelines, the Animal Welfare Act, and U.S. federal law.

### Bacterial strains and growth analysis.

S. aureus LAC strain was obtained from the Network on Antimicrobial Resistance in Staphylococcus aureus (NARSA). The S. aureus LAC strain lacking the *hptARS* and *uhpT* genes (ΔHpt) and the complemented strain were constructed in our previous study (27). S. aureus strains were cultured at 37°C for 24 h in CDM (40) supplemented with 0.2% (wt/vol) of each sugar (glucose, maltose, fructose, G6P, mannose, trehalose, or mannitol) as the sole carbon source. Bacterial growth was analyzed by measuring the optical density at 600 nm using a Cytation 5 reader (BioTek).

### Exoprotein preparation and immunoblot analysis.

S. aureus strain LAC was grown in CDM as described above, and cultures were normalized by optical density to synchronize the cultures to have consistent numbers of bacteria. Exoproteins were partially purified by trichloroacetic acid (TCA) precipitation. Protein samples were separated on 12.5% SDS-PAGE gels and then either stained with Coomassie blue or transferred to polyvinylidene difluoride (PVDF) membranes for immunoblotting. Membranes were blocked with 5% skim milk, immunoblotted with mouse anti-Hla monoclonal antibody (IBT Bioservices) and polyclonal chicken primary antibodies specific for the S component of leukocidins (HlgA, HlgC, LukE, and LukS), the F component of leukocidins (HlgB, LukD, and LukF), SElQ, SElK, and SElX generated using the chicken IgY service (Aves Laboratories), and detected with horseradish peroxidase (HRP)-conjugated anti-chicken IgY secondary antibody (GE Healthcare) and chemiluminescent substrate (Pierce).

### Cytotoxicity assay.

Human whole leukocytes were isolated by lysing erythrocytes with ammonium-chloride-potassium lysis buffer (Gibco). Human neutrophils were isolated by density gradient centrifugation using the Polymorphprep kit according to the manufacturer’s instructions (Axis-Shied). Cells were subsequently washed with phosphate-buffered saline (PBS) and resuspended in phenol red-free RPMI 1640 medium supplemented with 2% fetal bovine serum (Gibco). For cytotoxicity assays, whole leukocytes were dispensed at 2 × 10^5^ cells/well in a final volume of 100 μl/well in a 96-well plate. Exoprotein samples described above were added to the cells and incubated for up to 1 h at 37°C. PI (10 μg/ml) was added, and the cells were further incubated for 15 min. The cytotoxicity was measured by the fluorescence signal at 590 nm using a NovoCyte flow cytometer (Agilent Technologies) and a Cytation 5 reader (BioTek). For Transwell experiments, human HEp2 cells were cultured in 24-well plates to 80% confluence. A 0.45-μm Transwell insert (Corning) containing human neutrophils at 1 × 10^5^ cells/well was placed in the 24-well plate. In some experiments, LukF and LukS (1 μg of each; IBT Bioservices) were added to the Transwells and incubated for up to 12 h. The cytotoxicity was monitored using a Cytation 5 reader (BioTek).

### qRT-PCR.

To measure transcriptional changes in the staphylococcal cytotoxin genes (*hla*, *hlgA*, *hlgB*, *hlgC*, *lukF*, *lukS*, *lukD*, *lukE*, *lukG*, and *lukH*), total RNA was extracted from S. aureus cells that had been cultured for 9 h at 37°C in CDM supplemented with each sugar, as described above, using an RNeasy extraction kit according to the manufacturer’s instructions (Qiagen). A total of 100 ng total RNA was used to perform qRT-PCR using a Superscript one-step qRT-PCR kit (Invitrogen), the primers listed in Table S1 in the supplemental material, and a QuantStudio 3 system (Applied Biosystems). The data were normalized by calculating the threshold cycle (*C_T_*) of the target minus the *C_T_* of the internal control (*gyrA*) (Δ*C_T_*). Relative quantification of the target gene was performed by the comparative *C_T_* method (ΔΔ*C_T_*).

### Quantification of G6P.

THP-1 cells (1 × 10^7^) and tissues samples from diabetic TALLYHO/JngJ mice and nondiabetic SWR/J mice were sonicated in ice-cold PBS. Cell lysates were centrifuged at 13,000 × *g* for 10 min and deproteinized with a 10-kDa-cutoff spin filter (Sigma-Aldrich). Intracellular G6P concentrations were quantified using a G6P assay kit (Sigma-Aldrich) according to the manufacturer’s instructions.

### Murine subcutaneous infection.

Male TALLYHO/JngJ and SWR/J mice were purchased from the Jackson Laboratory at the age of 9 weeks and were allowed to acclimate for 1 week. Blood glucose levels were monitored using an AlphaTrak2 monitoring system (Zoetis). When TALLYHO/JngJ mice naturally developed hyperglycemia, typically at the age of 12 weeks, the animals were anesthetized and shaved from the dorsum. Diabetic TALLYHO/JngJ mice and nondiabetic SWR/J mice (*n* = 4/strain in two independent experiments) were subcutaneously infected with 10 μl of PBS containing 1 × 10^6^ CFU of wild-type, ΔHpt, or complemented S. aureus strain LAC. After 10 days, the animals were humanely euthanized, and the areas of skin lesions were measured using a SilhouetteStar camera and accompanying SilhouetteCentral data analysis software (ARANZ Medical). Skin lesions were homogenized, and serial dilutions were plated on blood agar plates to enumerate the bacteria existing in the skin lesions.

### Statistical analysis.

The statistical significance of data from different treatment groups was analyzed by Student's *t* test for simple comparison of two groups and analysis of variance (ANOVA) and Tukey’s multiple-comparison test for multiple comparisons using GraphPad Prism (*P* < 0.05).

### Data and resource availability.

All data generated or analyzed during this study are included in the main text and supplementary table. Additional data related to this article are available from the corresponding author upon reasonable request.
