# Supplementary material for: Role of Glucose-6-Phosphate in Metabolic Adaptation of Staphylococcus aureus in Diabetes
Source: Microbiol Spectr. 2021 Sep 22;9(2):e00857-21. doi: 10.1128/Spectrum.00857-21 (PMC8557822; doi:10.1128/Spectrum.00857-21)
Supplement: SUPPLEMENTAL FILE 1 — Supplemental material. Download SPECTRUM00857-21_Supp_1_seq8.pdf, PDF file, 0.1 MB [file spectrum00857-21_supp_1_seq8.pdf]

Supplemental table 1. Primers used in the study

| Primer | Sequences (5'->3') |
|---|---|
| RT Hla F | GTTTAGCCTGGCCTTCAGCC |
| RT Hla R | TTTTGTATCAATCGAATTTCTTGGAT |
| RT HlgA F | TTTGCACAAGACCCAACTGG |
| RT HlgA R | ATCTGGGACGAAATAGTCTCGTG |
| RT HlgB F | TCACACAGACAAGATGGCGC |
| RT HlgB R | CCTGCCCAGTAGAAGCCATTC |
| RT HlgC F | AAAAGATCAACGCATTATGGCA |
| RT HlgC R | TTCCAATTGACCTCGTATTTCACA |
| RT LukF F | CGAAAACAAAACGCTGCAAA |
| RT LukF R | TTACCTATCCAGTGAAGTTGATTCCA |
| RT LukS F | AGCTGCAACATTGTCGTTAGGA |
| RT LukS R | CCTCAGCGCCATCACCA |
| RT LukD F | GGATCAAAATCATTTTCAGAAACGA |
| RT LukD R | TCAACACCCCAGCCAATTG |
| RT LukE F | CAAAGTCCAAATGGTCCAACAG |
| RT LukE R | GTGATAAACGATGGATTAAAGCCA |
| RT LukG F | TTTGCACCAAAAAATCAGGATG |
| RT LukG R | TTTCCAGTTAAGCCTCCACGAT |
| RT LukH F | CAAAAGGTATTGGACGAACTTCATC |
| RT LukH R | CCAGTTATTATTTTTACCGCTGGC |
| RT GyrA F | CGTGAAGGTGACGAAGTTGTAGG |
| RT GyrA R | TAACTGGCGTACGTTTACCATAAC |
